# Transformation and survival in marginal zone lymphoma: a Finnish nationwide population-based study

**DOI:** 10.1038/s41408-023-00831-9

**Published:** 2023-04-25

**Authors:** Ilja Kalashnikov, Tomas Tanskanen, Leevi Viisanen, Nea Malila, Sirkku Jyrkkiö, Sirpa Leppä

**Affiliations:** 1 Research Program Unit, Applied Tumor Genomics Research Program, Faculty of Medicine, University of Helsinki, Helsinki, Finland; 2 Department of Oncology, Helsinki University Hospital Comprehensive Cancer Centre, Helsinki, Finland; 3 iCAN Digital Precision Cancer Medicine Flagship, Helsinki, Finland; 4 Finnish Cancer Registry, Cancer Society of Finland, Helsinki, Finland; 5 Department of Oncology and Radiotherapy, Turku University Hospital, University of Turku, Turku, Finland

**Keywords:** B-cell lymphoma, Epidemiology

## Abstract

Marginal zone lymphoma (MZL) is an indolent B-cell malignancy with heterogeneous anatomical and clinical presentation. While MZLs are generally associated with long survival, some patients experience histological transformation to aggressive large B-cell lymphoma. Population-based long-term data on the transformation of MZL is limited. We conducted a nationwide population-based study to estimate the risk of transformation and relative survival in patients diagnosed with MZL in Finland from 1995–2018. We identified a total of 1454 patients with MZL from the Finnish Cancer Registry (FCR). The cumulative incidence of transformation was 4.7% (95% CI, 3.6−6.2) at 10 years. The highest incidence of transformation was observed in the patients with splenic MZL (14.0%; 95% CI, 8.6−22.7). The transformation was associated with a substantially increased risk of death (HR, 5.18; 95% CI, 3.58–7.50). Ten-year relative survival was 79% (95% CI, 73‒83%). Transformation, nodal MZL subtype, and older age at diagnosis were associated with increased excess mortality, whereas patients diagnosed at a later calendar period had a lower excess risk of death. We conclude that transformation resulted in a substantially increased mortality irrespective of MZL subtype compared with the patients without transformation. Our results also suggest a reduction in excess mortality in recent years.

## Introduction

Marginal zone lymphoma (MZL) is a rare B-cell malignancy with unique clinicopathological features accounting for 5−15% of all new mature B-cell lymphoma cases in Western countries [1–3]. MZL mainly affects persons in the sixth and seventh decade of life with no notable sex dominance [2, 3]. The incidence varies between 0.5–2.5 per 100,000 depending on the geographic region and an increased trend in incidence has been observed during the last two decades [4–7].

Two recently updated classifications recognize three distinct MZL entities depending primarily on the initial site of lymphoma involvement: extranodal MZL of mucosa-associated lymphoid tissue (EMZL), splenic (SMZL), and nodal (NMZL) [2, 3]. EMZL comprises about 70% of all MZL cases and has been described in virtually all tissues, although the stomach is the most common primary site. SMZL and NMZL are rare and account for ~20 and 10% of all MZLs, respectively. The risk of MZL is strongly associated with several autoimmune diseases, infectious agents, and chronic inflammation [2, 3].

Although MZL entities differ in regard to biology, clinical presentation, and management, they are generally associated with long survival [2, 3, 8]. However, irrespective of the MZL subtype, a subset of patients experience transformation to aggressive large B-cell lymphoma and have increased mortality [2, 3, 9]. Several prognostic indexes have been developed to identify patients with more aggressive diseases, but none of them are significant predictors of transformation [10].

Population-based data on transformation in patients with MZL is limited since most cancer registries do not capture transformation status. Therefore, the current literature is mainly comprised of retrospective studies using databases from larger institutions, which may not be representative of the general population [10–13]. The results regarding the risk of transformation across MZL subtypes are also inconclusive [10, 13].

We conducted a nationwide population-based study of MZL to estimate the risk of transformation to large B-cell lymphoma and studied relative survival in patients diagnosed with MZL in Finland from 1995−2018. We also compared excess mortality between MZL patients with and without transformation.

## Methods

### Data sources

The Finnish Cancer Registry (FCR) is a statistical and epidemiological research institute responsible for registering nationwide data on all incident cancer diagnoses in Finland since 1953. Based on special legislation, physicians, hospitals, and pathology and hematology laboratories are obliged to report cancer cases to the FCR without the consent of the patient. The reports include a unique personal identity code, which allows for linkage between national registries and a reliable follow-up of cancer patients. Extensions, recurrences, or metastases of previously recorded primary cancers are reported to the FCR but not registered as separate cases. However, transformed cases are registered as new entities and can therefore be identified in patients with another hematological malignancy earlier. Since 2007, the coding of cancer cases has followed the International Classification of Diseases for Oncology, 3rd Edition (ICD-O-3), which is consistent with the World Health Organization (WHO) Classification of Tumors of Haematopoietic and Lymphoid Tissues, 4th edition [14, 15]. In 1953–2006, cancer morphology was coded according to a slightly modified version of the Manual of Tumor Nomenclature and Coding. In 2007, former codes were converted to ICD-O-3 [16, 17]. Information regarding the vital status and place of residence of cancer patients is obtained continuously from the Population Information System maintained by the Digital and Population Data Services Agency. Causes of death for cancer patients are received from Statistics Finland once per year. The FCR has high coverage overall, and in a recent study, the completeness for non-Hodgkin lymphomas was estimated at 94%, and 99% of cases were morphologically verified [18, 19].

### Patients

Patients diagnosed with incident MZL not otherwise specified (ICD-O-3 morphology code 9699/3; comprising EMZL and NMZL entities) and incident SMZL (ICD-O-3 morphology code 9689/3) in Finland in 1995–2018 were retrieved from the FCR database. In addition, patients diagnosed with lymphoma not otherwise specified (ICD-O-3 morphology code 9591/3 or 9590/3) in 1995–2006 were reviewed manually from the original pathology reports and recoded using ICD-O-3. From these cases, MZLs were also retrieved from the same calendar period.

Patient selection was restricted to those diagnosed as of 1995 to ensure a representative cohort considering that the distinction between MZL subtypes was first introduced in the revised European American lymphoma (REAL) classification of 1994 [20].

A total of 1637 patients were initially identified. The diagnosis and subtype of MZL and possible transformation were confirmed from the free-text part of the pathology reports. MZL was classified as EMZL, SMZL, or NMZL according to the WHO classification [14]. EMZL was further subclassified according to the primary anatomical site of involvement into gastric and non-gastric EMZL. Patients for whom the pathology report did not state a specific MZL subtype were termed unclassifiable. Clinical cancer notification reports were used for additional confirmation.

We excluded 76 patients with incorrect registry entries, or unclear or missing cancer notifications and 39 patients diagnosed with another lymphoma before MZL. Fifty-one patients diagnosed concurrently with a combination of MZL and a large B-cell lymphoma, i.e., composite lymphoma, and 17 patients identified through death certificate or autopsy only were also excluded from the study.

The study population consisted of 1454 patients after the above-mentioned exclusions. The incident transformation was defined as the diagnosis of a morphologically verified large B-cell lymphoma or high-grade B-cell lymphoma at least 3 months after the primary diagnosis of MZL. None of the transformations were registered by death certificate or autopsy only.

The statistical underlying cause of death for deceased MZL patients was retrieved from Statistics Finland. Cause of death was determined according to the selection and application rules of the International Classification of Diseases, 10th revision, compiled by the WHO [21].

For all patients, we retrieved data on sex, date of birth, date of diagnosis, date of last follow-up, vital status at the end of follow-up, possible date of transformation, and underlying cause of death. The study cohort was followed until December 31, 2018. No patient was lost to follow-up before the end of the study period. The general population mortality rates of Finland were obtained from Statistics Finland. Cause of death was categorized into three groups: any lymphoma, secondary malignant neoplasm, or other cause.

The study was approved by the National Institute for Health and Welfare (Dnro THL/1441/5.05.00/2019), Statistics Finland (Dnro TK-53-1172-19), and Helsinki University Hospital Institutional Review Board.

### Definitions and statistical analysis

The start of follow-up was defined as the date of MZL diagnosis. We used the Kaplan-Meier method to estimate overall survival. Hazard ratios (HRs) for total mortality and incident transformation were estimated using multivariable Cox regression models. The Aalen-Johansen estimator was used to estimate the cumulative incidence (i.e., risk) of transformation in MZL patients overall, considering death from other causes as a competing risk. To estimate cumulative incidence functions for patient subgroups, we used cause-specific Cox models for incident transformation and death from other causes.

Relative survival is defined as the ratio of the overall survival of patients to the expected survival of an equivalent group from the general population, matched to the patients by age, sex, and calendar period. Expected survival was estimated from Finnish population life tables. Relative survival was estimated using the Ederer II method with internal age standardization (age groups: 0−44, 45−54, 55−64, 65−74, and ≥75 years) [22]. Follow-up time was split into monthly intervals. Age-specific relative survival was estimated for three age groups by age at diagnosis (0−54, 55−74, and ≥75 years). A complete analysis was based on all person-time and deaths in 1995−2018, and period analyses were carried out for 1995−2006 and 2007−2018, separately, with left-truncated data for the later period [23].

Excess mortality is defined as the difference between the mortality rate observed in a population of cancer patients and the mortality rate of a comparable group from the general population. To estimate HRs for excess mortality, we used multivariable flexible parametric survival models [24]. Expected mortality was based on the general population mortality rates of Finland by 1-year age group, calendar year, and sex. The baseline excess hazard rate was modeled with 4 degrees of freedom [25].

Transformation cannot be measured at baseline. Therefore, to account for immortal time bias, the transformation was modeled as a time-varying covariate that may change during follow-up. To allow for non-proportional hazards, we fit separate models that also included time from transformation. After splitting the follow-up time into monthly intervals, the time-dependent effect of transformation was modeled with a restricted cubic spline with 2 degrees of freedom.

The Wilcoxon rank-sum and Kruskal–Wallis tests were used to compare age at diagnosis between patient groups.

Statistical analyses were performed using R, version 4.0.2 (R Foundation for Statistical Computing, Vienna, Austria), with the packages survival 3.1-12, rstpm2 1.5.2, Epi 2.44, and popEpi 0.4.8.

## Results

### Patients

Baseline characteristics of 1454 patients, as well as subtype-specific rates of transformation and mortality, are shown in Table 1. The median age at diagnosis was 68 years for all MZL subtypes together (interquartile range (IQR), 58−77; range, 13–95), and there was a slight female predominance (59%).

**Table 1:** Characteristics of patients diagnosed with marginal zone lymphoma in Finland, 1995–2018.

MZL subtype	No. of patients (%)	Median age at diagnosis (IQR)	Male/female	Median year of diagnosis (IQR)	Median follow-up, years (IQR)	Follow-up time, person-years	No. of deaths (%)	Mortality rate (per 1000 person-years)	No. of transformations (%)	Transformation rate (per 1000 person-years)
Non-gastric EMZL	746 (51.3)	67.5 (56.3−76.3)	292/454	2009 (2003−2015)	5.8 (2.4−11.0)	5479	250 (45.0)	45.6	20 (36.4)	3.7
Gastric EMZL	324 (22.3)	70.3 (60.1−77.2)	148/176	2005 (1999−2011)	6.6 (2.1−12.4)	2590	175 (31.5)	67.6	8 (14.5)	3.1
SMZL	186 (12.8)	67.9 (59.6−75.5)	68/118	2012 (2008−2016)	4.4 (2.1−8.5)	1069	60 (10.8)	56.1	16 (29.1)	15.7
NMZL	91 (6.3)	67.7 (61.5−74.2)	44/47	2015 (2010−2017)	2.5 (1.1−5.1)	346	28 (5.0)	80.8	6 (10.9)	17.8
Unclassifiable	107 (7.4)	70.8 (60.2−79.5)	42/65	2012 (2005−2016)	4.4 (1.8−7.7)	589	42 (7.6)	73.3	5 (9.1)	8.6
All subtypes	1454 (100.0)	68.2 (58.1−76.5)	594/860	2009 (2003−2015)	5.4 (2.0−10.4)	10073	555 (100.0)	55.1	55 (100.0)	5.5

EMZL was the most frequent subtype, with 1070 cases (74%), followed by SMZL (186; 13%) and NMZL (91; 6.3%) cases, respectively. The MZL subtype could not be established in 107 (7.4%) of the cases. There were no clinically significant differences in median age at diagnosis between the males and females or in the MZL subtypes. The median follow-up time was overall 5.4 years (IQR, 2.0−10.4; range, 0‒24 years).

The most common primary site for EMZL was the stomach (*n* = 324; 30%), followed by the eye (*n* = 182; 17%), salivary gland (*n* = 140; 13%), and lung (*n* = 113; 11%).

### Risk of transformation

**Fig. 1:**
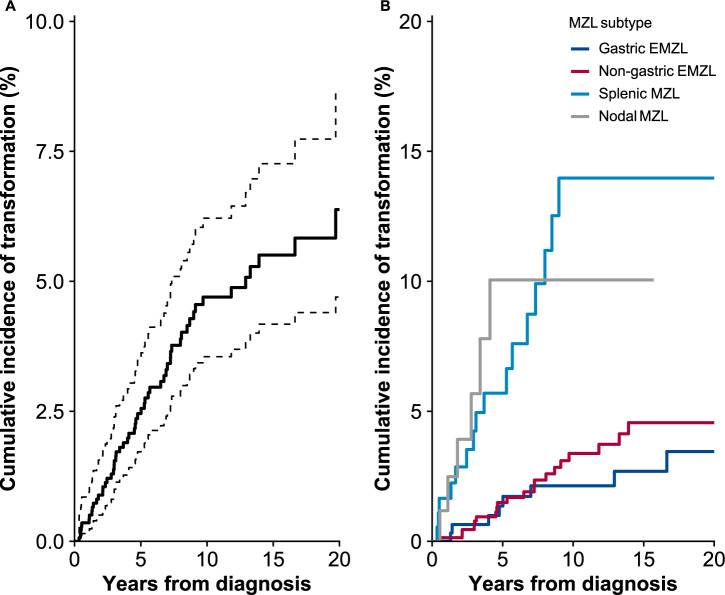
Cumulative incidence of transformation in patients diagnosed with marginal zone lymphoma in Finland in 1995–2018. **A** Cumulative incidence of transformation for the whole cohort with 95% confidence intervals; **B** Cumulative incidence of transformation stratified by marginal zone lymphoma subtype. Estimates for unclassifiable subtypes are not shown. MZL marginal zone lymphoma, EMZL extranodal marginal zone lymphoma.

An incident transformation occurred in 55 MZL patients during 9931 person-years of follow-up (crude transformation rate, 5.5/1000). The cumulative incidence of transformation for the entire cohort was 2.5% (95% CI, 1.7−3.5) at 5 years and 4.7% (95% CI, 3.6−6.2) at 10 years from diagnosis (Fig. 1A). Majority of the transformations (49 of 55; 89%) occurred within 10 years from diagnosis; the remaining six (11%) occurred between 10 and 19 years. Thirty-one (56%) of the transformations occurred in females, with a crude transformation rate of 5.1/1000 compared to 6.2/1000 for males. The median age at the time of transformation was 69 years (IQR, 62–77; range, 53–89), with no clinically significant differences between males and females.

HRs for incident transformation by MZL subtype adjusted for sex, age, and year of diagnosis are shown in Table 2. Compared to the patients with gastric EMZL, the risk of transformation was significantly increased in patients with SMZL (HR, 4.43, 95% CI, 1.83–10.8; *p* < 0.001) and NMZL (HR, 4.71, 95% CI, 1.55–14.3; *p* = 0.006) but not significantly different in the patients with non-gastric EMZL (HR, 1.16; 95% CI, 0.50–2.67; *p* = 0.7) or MZL with unclassifiable subtype (HR 2.44; 95% CI 0.78–7.64; *p* = 0.13) (Table 2). The risk of transformation did not differ significantly by age at diagnosis (HR, 1.10 per 10-year increase; 95% CI, 0.88–1.38; *p* = 0.4), sex (HR, 0.76 for females compared with males; 95% CI, 0.44–1.30; *p* = 0.3), or year of diagnosis (HR, 1.61 per 10-year increase; 95% CI, 0.91–2.83; *p* = 0.1).

**Table 2:** Hazard ratios for incident transformation in patients with marginal zone lymphoma.

Characteristic	HR^a^	95% CI^b^	*p* value
Age at diagnosis (per 10-year increase)	1.1	0.88−1.38	0.4
Sex			
Male	1	−	−
Female	0.76	0.44−1.30	0.3
MZL subtype			
Gastric EMZL	1	−	−
Non-gastric EMZL	1.16	0.50−2.67	0.7
SMZL	4.43	1.83−10.8	<0.001
NMZL	4.71	1.55−14.3	0.006
Unclassifiable	2.44	0.78−7.64	0.13
Year of diagnosis (per 10-year increase)	1.61	0.91−2.83	0.1

^a^HR hazard ratio.

^b^CI confidence interval.

After adjustment for age, sex and MZL subtype, we did not find a significant association between the period of diagnosis and the risk of transformation (HR, 1.43 for 2003‒2018 compared with 1995‒2002; 95% CI, 0.71‒2.88, *p* = 0.3).

The cumulative incidence of transformation at 10 years from diagnosis was 14.0% in patients with SMZL (95% CI, 8.6−22.7), 10.1% in NMZL (95% CI, 4.6−22.0), and 5.4% in unclassifiable MZL subtype (95% CI, 2.0−14.4). The cumulative incidence of transformation at 10 years was higher in patients with non-gastric EMZL (3.4%; 95% CI, 2.1−5.4) than in patients with gastric EMZL (2.1%; 95% CI, 1.0−4.7) (Fig. 1B).

**Fig. 2:**
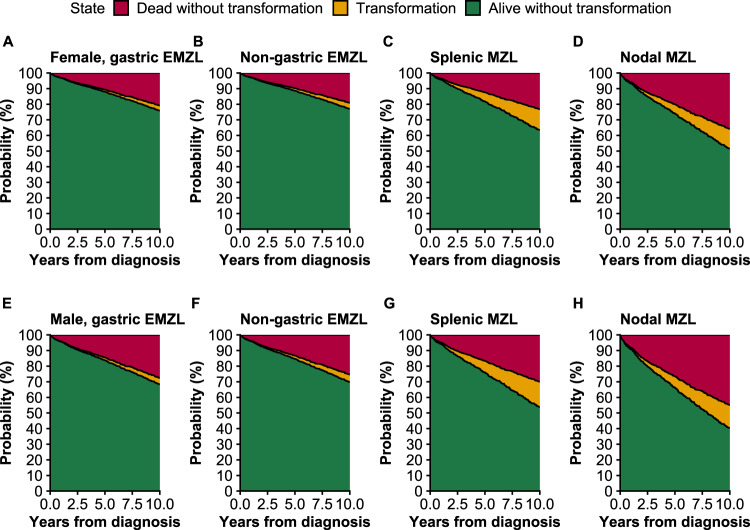
Stacked cumulative incidence curves for patients diagnosed with marginal zone lymphoma (MZL) at age 65 years in Finland in 2008. **A** Female, gastric extranodal marginal zone lymphoma (EMZL); **B** female, non-gastric EMZL; **C** female, splenic MZL; **D** female, nodal MZL; **E** male, gastric EMZL; **F** male, non-gastric EMZL; **G** male, splenic MZL; **H** male, nodal MZL.

Figure 2 shows the sex- and subtype-specific cumulative incidence probabilities for patients diagnosed with MZL in 2008, at age 65. Males and females with EMZL (male, 0.69; 95% CI, 0.65−0.74; female, 0.77; 95% CI, 0.73−0.80) had a higher probability of being alive without transformation compared to patients with NMZL subtype (male, 0.40; 95% CI, 0.28−0.57; female, 0.51; 95% CI, 0.40−0.66). Moreover, the probability of transformation was increased in patients with NMZL (male, 0.15; 95% CI, 0.07−0.30; female, 0.13; 95% CI, 0.06−0.27) and SMZL (male, 0.16; 95% CI, 0.10−0.27; female, 0.13; 95% CI, 0.08−0.22) compared to EMZL (male, 0.05; 95% CI, 0.03−0.07; female, 0.04; 95% CI, 0.02−0.06). Finally, the probability of death without transformation was higher in NMZL (male, 0.45; 95% CI, 0.34−0.60; female, 0.36; 95% CI, 0.26−0.49) than in other subtypes.

### Overall survival

There were 555 deaths during 10073 person-years of follow-up (crude mortality rate, 55.1/1000). The overall survival probability was 76% (95% CI, 74–78%) at 5 years and 59% (95% CI, 56–62%) at 10 years from diagnosis.

Among the 55 patients with transformation, 32 died during 142 years of follow-up after transformation, with a crude mortality rate of 225/1000. Five hundred twenty-three deaths occurred in patients without transformation during 9930 person-years of follow-up (crude mortality rate, 52.7/1000).

**Fig. 3:**
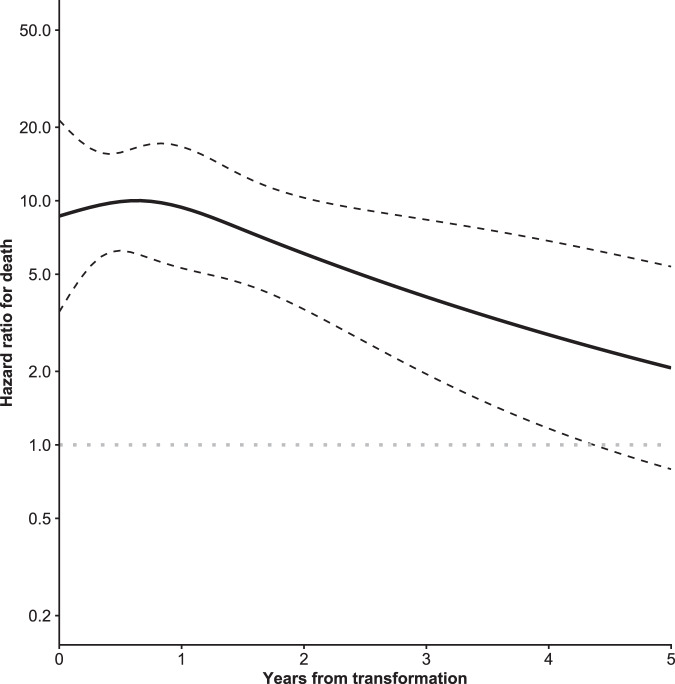
Hazard ratio for death (on a logarithmic scale on the y-axis) by the time from transformation. The dashed lines indicate 95% confidence intervals.

After adjustment for age, sex, MZL subtype, and year of diagnosis, the transformation was associated with a substantial increase in total mortality (HR, 5.18; 95% CI, 3.58–7.50; *p* < 0.001) (Table 3). The relative risk of death, as compared with the patients without transformation, was highest soon after transformation (Fig. 3). The time-dependent HRs at 1 and 5 years after transformation were 9.40 (95% CI, 5.30‒16.7) and 2.07 (95% CI, 0.80‒5.38), respectively. Furthermore, NMZL was associated with increased total mortality as compared to gastric EMZL (HR, 1.94; 95% CI, 1.28–2.95; *p* = 0.002). We did not observe any differences in the effect of transformation on total mortality between the MZL subtypes (*p* = 0.7).

**Table 3:** Hazard ratios for total and excess mortality in patients with marginal zone lymphoma.

	Total mortality			Excess mortality		
Characteristic	HR^a^	95% CI^b^	*p* value	HR^a^	95% CI^b^	*p* value
Age at diagnosis (per 10-year increase)	2.48	2.26−2.72	<0.001	1.95	1.63−2.33	<0.001
Sex						
Male	1	−	−	1	−	−
Female	0.7	0.59−0.84	<0.001	0.81	0.57−1.14	0.2
MZL subtype						
Gastric EMZL	1	−	−	1	−	−
Non-gastric EMZL	0.89	0.73−1.08	0.2	0.68	0.45−1.03	0.07
SMZL	1.18	0.87−1.60	0.3	1.08	0.61−1.93	0.8
NMZL	1.94	1.28−2.95	0.002	2.35	1.24−4.48	0.009
Unclassifiable	1.33	0.94−1.88	0.11	1.46	0.79−2.69	0.2
Year of diagnosis (per 10-year increase)	0.6	0.51−0.70	<0.001	0.44	0.33−0.60	<0.001
Transformation						
Nontransformed	1	−	−	1	−	−
Transformed	5.18	3.58−7.50	<0.001	14	8.44−23.2	<0.001

^a^HR hazard ratio.

^b^CI confidence interval.

### Relative survival

**Fig. 4:**
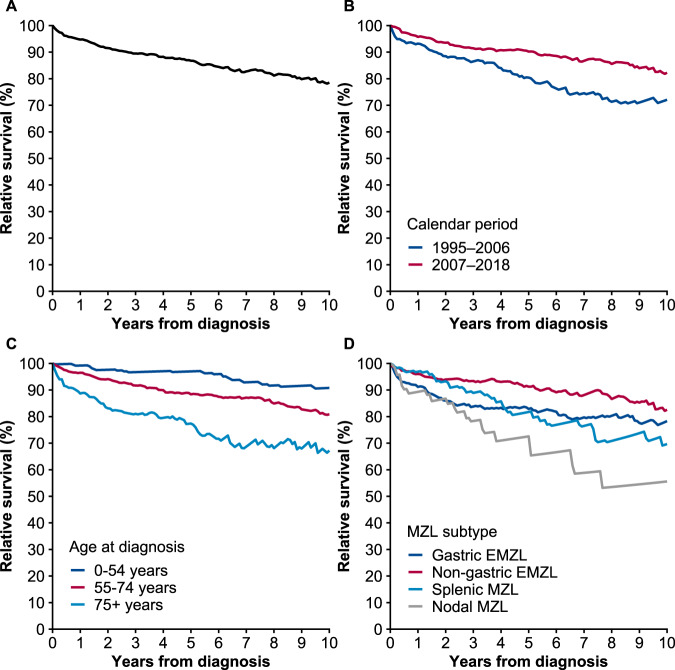
Relative survival in patients diagnosed with marginal zone lymphoma in Finland in 1995–2018. **A** Ten-year age-standardized relative survival for 1995–2018; **B** Ten-year age-standardized relative survival for 1995–2006 and 2007–2018; **C** Ten-year age-specific relative survival; **D** Ten-year age-standardized relative survival by subtype. MZL marginal zone lymphoma, EMZL extranodal marginal zone lymphoma.

Over the entire follow-up time, the 10-year age-standardized relative survival of MZL patients was 79% (95% CI, 73‒83%) (Fig. 4A). In the period analysis, the 10-year age-standardized relative survival was 82% (95% CI, 75‒88%) in 2007–2018 in comparison to 72% (95% CI, 61‒80%) in 1995‒2006 (Fig. 4B). Relative survival was lower in the patients diagnosed at the age of 75 years or older than in the younger patients (Fig. 4C). After stratification according to the MZL subtypes (excluding unclassifiable), the 10-year age-standardized relative survival was the highest in the patients with non-gastric EMZL (83%; 95% CI, 73–89), followed by the patients with gastric EMZL (78%; 95% CI, 67–86), SMZL (70%; 95% CI, 53–81), and NMZL (56%; 95% CI, 43– 67) (Fig. 4D).

Adjusted HRs for excess mortality in the entire cohort are shown in Table 3. Older age at diagnosis was associated with higher excess mortality (HR, 1.95 per 10-year increase; 95% CI, 1.63‒2.33; *p* < 0.001), whereas patients diagnosed at a later calendar year had a lower excess risk of death (HR, 0.44 per 10-year increase; 95% CI, 0.33‒0.60; *p* = <0.001). In addition, the NMZL subtype was associated with increased excess mortality in comparison to gastric EMZL (HR, 2.35; 95% CI, 1.24–4.48; *p* = 0.009). The transformation was associated with a substantial increase in excess mortality (HR, 14.0; 95% CI, 8.44‒23.2; *p* < 0.001), but the excess relative risk decreased over time. The time-dependent excess HRs at 1 and 5 years after transformation were 23.2 (95% CI, 11.5‒47.0) and 2.46 (95% CI, 0.22‒27.4), respectively. There was no association between sex and excess mortality. We did not observe any differences in the effect of transformation on excess mortality between the MZL subtypes (*p* = 0.6).

### Causes of death

Of the 555 deaths during the study period, 233 (42.0%) were due to any lymphoma, 72 (13.0%) to another secondary malignant neoplasm, and 250 (45.0%) to other causes. Other causes of death were most frequently related to cardiovascular diseases. Subtype-specific causes of death are shown in Table 4. Of the 32 deaths recorded in the patients with incident transformation, 29 (91%) were due to lymphoma.

**Table 4:** Causes of death in patients with marginal zone lymphoma.

MZL subtype	Any lymphoma (%)	Secondary malignant neoplasm (%)	Other cause (%)	Total (%)
Non-gastric EMZL	105 (42.0)	28 (11.2)	117 (46.8)	250 (100)
Gastric EMZL	62 (35.4)	33 (18.9)	80 (45.7)	175 (100)
SMZL	35 (58.3)	6 (10.0)	19 (31.7)	60 (100)
NMZL	15 (53.6)	3 (10.7)	10 (35.7)	28 (100)
Unclassifiable	16 (38.1)	2 (4.8)	24 (57.1)	42 (100)

## Discussion

In this large nationwide, population-based cohort of patients diagnosed with MZL in Finland in 1995–2018, the risk of transformation for the entire cohort was 4.7% at 10 years. The highest risk was observed in the SMZL and NMZL subtypes. The transformation resulted in a substantially increased mortality irrespective of MZL subtype in comparison to patients without transformation. Patients with NMZL were also more likely to die without transformation as compared to those with other MZL subtypes. We found that the 10-year relative survival for the entire study period was 79% with better survival rates in the non-gastric and gastric EMZL subtypes as compared to SMLZ and NMZL. Our results indicate a reduction in excess mortality in 1995–2018 and an improvement in 10-year relative survival between 1995–2006 and 2007–2018. Finally, excess mortality was higher in patients diagnosed at an older age.

Consistent with the previous studies, EMZL was the most common subtype, followed by SMZL and NMZL [2, 3, 26]. In addition, the proportions of the primary site of involvement within the EMZL subtype are in line with another large study [8]. Interestingly, in a study from the United States, approximately one-third of all MZL cases were classified as NMZL [4]. Importantly, in the US study, the NMZL subtype was not defined according to the WHO classification but rather on the primary site of involvement without consideration of possible extranodal or splenic involvement [4, 14]. The MZL subtype distribution of the US study is, therefore, not comparable to our study.

In general, MZL is associated with an indolent clinical course and relatively low excess mortality [2, 3, 8, 27]. Consistent with another large study [8], the 10-year relative survival was 79% in our study, and the patients with non-gastric EMZL had the highest relative survival compared to other MZL subtypes. In addition, we observed that 10-year relative survival improved between 1995−2006 and 2007−2018. We found that the leading cause of death in MZL patients was due to other causes (45.0%), followed by any lymphoma (42.0%) and another secondary malignant neoplasm (13.0%). Notably, lymphoma was the major cause of death in patients with incident transformation (91%). While a recent prospective study on the causes of death in low-grade B-cell lymphomas reported a lower proportion of lymphoma-related mortality in MZL, it is important to note that direct comparison with our study is not feasible due to differences in patient population, study design, calendar period of enrollment, and definition of causes of death [28]. Furthermore, a high proportion of unknown causes of death in that study may explain the reported lower lymphoma-related mortality [28]. Taken together, our findings suggest that lymphoma remains a significant cause of mortality in MZL patients, especially those with incident transformation.

The risk of transformation for the entire cohort was 4.7% (95% CI, 3.6−6.2) at 10 years. The highest risk was observed in the patients with SMZL and NMZL compared to EMZL. Interestingly, patients with NMZL were also more likely to die without transformation as compared to those with other MZL subtypes. A large retrospective single-center study from the United States comprising 453 patients reported a 10-year risk of transformation of 8.4% based on a competing risk approach [10]. Consistent with our findings, the risk of transformation was higher in SMZL and NMZL subtypes [10]. In another retrospective study conducted by Conconi et al., the transformation occurred in 13 of 340 patients diagnosed between 1995−2012 (3.8%). However, the risk of transformation was not estimated for a defined time interval [13]. The majority of the previous studies have been limited either by the lack of population-based data collection (single-institution or pooled multi-center studies), small sample size, exclusion of certain MZL subtypes, short follow-up time, or lack of histological verification of transformation [10–13, 29].

We excluded 51 patients with composite lymphoma from our cohort. However, we cannot exclude the possibility that these patients had an undiagnosed MZL, which eventually transformed into composite lymphoma. Conversely, composite lymphoma may have developed as a primary neoplasm.

The total mortality was substantially higher in patients with transformation compared with patients without transformation. To account for immortal time bias, the transformation was considered as a time-varying covariate [30]. One explanation for the decline in relative risk of death over time could be that patients who have survived a certain period after transformation represent a selected population with less aggressive disease, better treatment response, or otherwise more favorable prognostic factors as compared to the overall population of patients with transformation.

Older age at diagnosis was associated with higher excess mortality. Excess mortality is a measure of disease- and treatment-related mortality, whereas total mortality is strongly influenced by causes unrelated to MZL. A possible explanation for higher excess mortality in older patients may be treatment-related toxicity, but the biology of MZL can also differ between age groups. There was no significant association between sex and excess mortality, which is in accordance with a large study from the United States [27].

We found a significant reduction in both total and excess mortality in patients diagnosed with MZL in more recent years. This can be explained at least partially by less aggressive treatment approaches and the introduction of biological agents in both up-front and recurrence settings. In addition, higher biopsy rates at recurrence in more recent years may have resulted in better detection of incident transformations with the adequate treatment given accordingly. However, the risk of transformation was not associated with the period of diagnosis in our study. Taken together, these factors may have reduced both early and late treatment-related mortality over time.

Of note, the incidence of MZL appears to have increased in Finland and other countries in the last two decades [4–7]. However, it is unclear whether this represents a true rise or changes in diagnostic practices. Therefore, lower excess mortality in more recent years may be in part due to earlier detection of MZL.

The strengths of this study are the population-based design and the use of high-quality nationwide cancer registry data from a long calendar period of 24 years. Furthermore, all MZL diagnoses and transformations were reviewed from the original pathology reports at the Finnish Cancer Registry. However, some limitations require careful consideration. First, we could not perform a central pathology review to confirm the diagnoses of MZL. Second, a proportion of patients could not be assigned a specific MZL subtype due to insufficient data. Third, detailed individual-level data on treatment was unavailable. Although we expect that treatment trends in Finland follow international guidelines, which strongly recommend a biopsy confirmation prior to establishing a diagnosis of histologic transformation, we cannot exclude the possibility that some patients were treated for presumed transformation based solely on clinical findings. Finally, FCR did not have information on stage, performance status, or comorbidities. Collectively, our data underscore the importance of long-term follow-up, re-biopsy at the time of relapse, and minimization of the risks for late adverse effects associated with treatment.

In conclusion, the 10-year relative survival for the patients diagnosed with MZL in Finland from 1995–2018 was 79%. Excess mortality was lower among MZL patients diagnosed in more recent years. The risk of transformation to aggressive B-cell lymphoma was 4.7% at 10 years, with higher rates observed for SMZL and NMZL subtypes. The transformation was associated with a substantially increased risk of death irrespective of the MZL subtype.

## Data Availability

The datasets generated during and/or analysed during the current study are available from the corresponding author on reasonable request.

## References

[1] Zucca E, Arcaini L, Buske C, Johnson PW, Ponzoni M, Raderer M (2020). Marginal zone lymphomas: ESMO Clinical Practice Guidelines for diagnosis, treatment and follow-up. Ann Oncol.

[2] Alaggio R, Amador C, Anagnostopoulos I, Attygalle AD, Araujo IB, de O (2022). The 5th edition of the World Health Organization classification of haematolymphoid tumours: lymphoid neoplasms. Leukemia.

[3] Campo E, Jaffe ES, Cook JR, Quintanilla-Martinez L, Swerdlow SH, Anderson KC (2022). The International Consensus classification of mature lymphoid neoplasms: a report from the Clinical Advisory Committee. Blood.

[4] Khalil MO, Morton LM, Devesa SS, Check DP, Curtis RE, Weisenburger DD (2014). Incidence of marginal zone lymphoma in the United States, 2001-2009 with a focus on primary anatomic site. Br J Haematol.

[5] Dandoit M, Mounier M, Guy J, Petrella T, Girard S, Casasnovas R-O (2015). The heterogeneity of changes in incidence and survival among lymphoid malignancies in a 30-year French population-based registry. Leuk Lymphoma.

[6] van Leeuwen MT, Turner JJ, Joske DJ, Falster MO, Srasuebkul P, Meagher NS (2014). Lymphoid neoplasm incidence by WHO subtype in Australia 1982-2006. Int J Cancer.

[7] Smith A, Crouch S, Lax S, Li J, Painter D, Howell D (2015). Lymphoma incidence, survival and prevalence 2004-2014: sub-type analyses from the UK’s Haematological Malignancy Research Network. Br J Cancer.

[8] Olszewski AJ, Castillo JJ (2013). Survival of patients with marginal zone lymphoma: analysis of the surveillance, epidemiology, and end results database. Cancer.

[9] Casulo C, Friedberg J (2017). Transformation of marginal zone lymphoma (and association with other lymphomas). Best Pr Res Clin Haematol.

[10] Alderuccio JP, Zhao W, Desai A, Gallastegui N, Ramdial J, Kimble E, et al. Risk factors for transformation to higher-grade lymphoma and its impact on survival in a large cohort of patients with marginal zone lymphoma from a single institution. *J. Clin. Oncol.* 2018; JCO1800138. 10.1200/JCO.18.00138 30312133

[11] Meyer AH, Stroux A, Lerch K, Eucker J, Eitle J, Hohloch K (2014). Transformation and additional malignancies are leading risk factors for an adverse course of disease in marginal zone lymphoma. Ann Oncol J Eur Soc Med Oncol.

[12] Maeshima AM, Taniguchi H, Toyoda K, Yamauchi N, Makita S, Fukuhara S (2016). Clinicopathological features of histological transformation from extranodal marginal zone B-cell lymphoma of mucosa-associated lymphoid tissue to diffuse large B-cell lymphoma: an analysis of 467 patients. Br J Haematol.

[13] Conconi A, Franceschetti S, Aprile von Hohenstaufen K, Margiotta-Casaluci G, Stathis A, Moccia AA (2015). Histologic transformation in marginal zone lymphomas†. Ann Oncol J Eur Soc Med Oncol.

[14] Swerdlow SH, Campo E, Harris NL, Jaffe ES, Pileri SA, Stein H, et al. WHO Classification of Tumours of Haematopoietic and Lymphoid Tissues. Revised 4th ed. Vol. 2. Lyon: International Agency for Research on Cancer; 2017.

[15] Fritz AG, editor. International classification of diseases for oncology: ICD-O. 3rd ed. First revision. Geneva: World Health Organization; 2013.

[16] World Health Organization. Manual of the international statistical classification of diseases, injuries, and causes of death based on the recommendations of the seventh revision Conference, 1955, and adopted by the ninth World Health Assembly under the WHO Nomenclature Regulations. (1957). https://apps.who.int/iris/handle/10665/42900.

[17] American Cancer Society. Manual of tumor nomenclature and coding. New York: American Cancer Society; 1951.

[18] Pukkala E, Engholm G, Højsgaard Schmidt LK, Storm H, Khan S, Lambe M (2018). Nordic Cancer Registries - an overview of their procedures and data comparability. Acta Oncol Stock Swed.

[19] Leinonen MK, Miettinen J, Heikkinen S, Pitkäniemi J, Malila N (2017). Quality measures of the population-based Finnish Cancer Registry indicate sound data quality for solid malignant tumours. Eur J Cancer Oxf Engl.

[20] Harris NL, Jaffe ES, Stein H, Banks PM, Chan JK, Cleary ML (1994). A revised European-American classification of lymphoid neoplasms: a proposal from the International Lymphoma Study Group. Blood.

[21] Official Statistics of Finland (OSF). Causes of death [e-publication]. ISSN=1799-5078. Helsinki: Statistics Finland [referred: 5.3.2023]. http://www.stat.fi/til/ksyyt/ksyyt_2021-10-29_luo_001_en.html.

[22] Ederer F, Heise H . Instructions to IBM 650 programmers in processing survival computations*.* Methodological note no. 10. Bethesda: National Cancer Institute; 1959.

[23] Brenner H, Gefeller O (1996). An alternative approach to monitoring cancer patient survival. Cancer.

[24] Nelson CP, Lambert PC, Squire IB, Jones DR (2007). Flexible parametric models for relative survival, with application in coronary heart disease. Stat Med.

[25] Syriopoulou E, Mozumder SI, Rutherford MJ, Lambert PC (2019). Robustness of individual and marginal model-based estimates: a sensitivity analysis of flexible parametric models. Cancer Epidemiol.

[26] Cheah CY, Zucca E, Rossi D, Habermann TM (2022). Marginal zone lymphoma: present status and future perspectives. Haematologica.

[27] Cerhan JR, Habermann TM (2021). Epidemiology of marginal zone lymphoma. Ann Lymphoma.

[28] Tun AM, Khurana A, Mwangi R, Link BK, Wang Y, Feldman AL (2022). Causes of death in low-grade B-cell lymphomas in the rituximab era: a prospective cohort study. Blood Adv.

[29] Xing KH, Kahlon A, Skinnider BF, Connors JM, Gascoyne RD, Sehn LH (2015). Outcomes in splenic marginal zone lymphoma: analysis of 107 patients treated in British Columbia. Br J Haematol.

[30] van Walraven C, Davis D, Forster AJ, Wells GA (2004). Time-dependent bias was common in survival analyses published in leading clinical journals. J Clin Epidemiol.

